# Management of Mixed Warm/Cold Autoimmune Hemolytic Anemia: A Case Report and Review of Current Literature

**DOI:** 10.1155/2023/1381861

**Published:** 2023-07-24

**Authors:** Elliot C. Smith, Nabeel Kahwash, Siavash Piran

**Affiliations:** ^1^Division of Hematology, Department of Medicine, University of Toronto, Toronto, Ontario, Canada; ^2^Trillium Health Partners, Etobicoke, Ontario, Canada

## Abstract

**Background:**

Mixed warm/cold autoimmune hemolytic anemia (AIHA) is a rare diagnostic entity with limited therapeutic options. Previous literature has described the diagnostic difficulty in this pathology and the limited response rates to corticosteroids. Furthermore, there is limited evidence regarding the use of rituximab in this condition.

**Methods:**

Alongside our case report, we conducted a scoping review of case reports/case series describing mixed AIHA, their treatment, and clinical outcomes since 2000. Inclusion criteria included a confirmed diagnosis of mixed AIHA (confirmed warm antibodies and cold agglutinins based on DAT). *Case Summary/Results*. We present a case of mixed AIHA in an 83-year-old female presenting with extensive, bilateral pulmonary embolisms and left renal vein thrombosis. The patient underwent extensive workup with no identifiable provoking etiology. Initial treatment involved prednisone therapy was transitioned to rituximab upon diagnosis of mixed AIHA. The patient demonstrated a mixed response with stable hemoglobin and transfusion independence; however, with persistently elevated hemolytic indices following completion of rituximab treatment. Our literature review identified 16 articles; two were excluded for unavailable clinical details. The most commonly associated conditions included autoimmune conditions (*n* = 5, 26%) and lymphoproliferative disorders (*n* = 3, 12%). The most common treatment involved corticosteroids; seven studies involved the use of rituximab.

**Conclusion:**

Mixed AIHA represents a complex diagnosis and optimal management is not well established. Consistent with our case, recent literature suggests a promising response to rituximab and a limited response to steroid treatment. Given the limited literature, additional studies are required to elucidate optimal management of this unique pathology.

## 1. Introduction

Mixed warm/cold autoimmune hemolytic anemia (AIHA) is a rare diagnostic entity with limited therapeutic options. Previous literature has described the diagnostic difficulty in this pathology and a limited response to steroid therapy. Furthermore, there is limited evidence regarding the use of rituximab in this condition. In this case report, we describe a case of severe mixed warm and cold autoimmune hemolytic anemia with a complicated diagnosis which was treated with both steroids and rituximab with a clinical response evidenced by stability in hemoglobin and improvement in hemolytic indices. Further, we conducted a review of recent literature involving case reports and series of mixed warm/cold AIHA and their clinical courses.

## 2. Research Aim

- To highlight the diagnostic difficulties within mixed warm/cold autoimmune hemolytic anemia and use of rituximab as a management strategy within this rare pathology

## 3. Case Report

Our case involved an 83-year-old female with a past medical history significant for provoked deep vein thrombosis (DVT) following an ankle fracture, hypertension, and osteoporosis endorsing a period of worsening fatigue and exertional dyspnea over the preceding several weeks. Upon admission, the patient was found to have elevated liver enzymes as well as D-Dimer greater than 4000 *μ*g/L (<500 *μ*g/L) which prompted further workup for thromboembolic disease. Computed tomography angiography identified extensive bilateral pulmonary emboli and a large left renal vein thrombus. Doppler venous US of the legs identified a left-sided proximal DVT. In addition, the complete blood count (CBC) showed a polychromic and normocytic anemia with a hemoglobin of 59 g/L requiring red cell transfusion, leukocytes of 13.7 × 10^9^/L, platelets of 277 × 10^9^/L, and reticulocytes of 57 × 10^9^/L (23–90 × 10^9^/L). Further investigations demonstrated evidence of hemolysis including elevated lactate dehydrogenase (LDH) of 1347 U/L (<140 U/L), total and direct bilirubin of 37 *μ*mol/L and 15 *μ*mol/L, respectively (<20 *μ*mol/L and <5 *μ*mol/L), and decreased haptoglobin of less than 0.08 g/L (0.30–2.00 g/L). On the peripheral smear, there were marked spherocytes and mild Howell–Jolly bodies with significant agglutination.

Initial hemolysis workup included a Direct Antibody Test (DAT) which was invalid twice due to positive controls. Investigations revealed positive cold autoantibody at 4 degrees Celsius (°C) and negative ones at 37°C. Due to clinical suspicion of cold autoimmune hemolysis, the patient was subsequently started on folic acid supplementation, and cold avoidance was recommended. Secondary etiologies including infection were investigated including HIV, EBV, and hepatitis B serologies, all of which were negative as well as SARS-CoV-2 PCR. Malignancy workup including CT chest, abdomen, and pelvis did not reveal evidence of malignancy. A bone marrow biopsy was also performed, demonstrating erythroid hyperplasia without evidence of a lymphoproliferative disorder, and paroxysmal nocturnal hemoglobinuria markers were also sent and found to be negative.

Due to ongoing clinically significant hemolysis, the patient was started on a trial of prednisone at a dose of 1 mg/kg for four days. Unfortunately, the patient continued to have significant hemolysis demonstrated by ongoing transfusion requirements as well as elevated hemolytic markers, and additional investigations were undertaken. Due to ABO type discrepancies, samples were sent to our reference lab at Canadian Blood Services for further analysis. The patient's red cells were found to be reactive by immediate spin with different monoclonal ABO antisera (anti-A, anti-B, and anti-A, B) due to the presence of a cold autoantibody. The determination of the cold antibody titre was unable to be performed and therefore remained unknown in this case. The interference was not eliminated by the washing patient's red blood cell suspension three times with prewarmed saline and retesting. The valid ABO typing was obtained by using the prewarmed technique and incubation at 37°C for an hour. A valid DAT result was obtained from testing the patient's dithiothreitol (DTT)-treated cells. The patient's plasma was reactive with all papain-treated cells tested by the indirect antiglobulin testing including autocontrol. An acid eluate prepared from the patient's red cells was reactive with all cells tested by the gel method indicating the presence of a warm autoantibody. Cold autoabsorption with rabbit erythrocytes stroma was also performed to remove the cold autoantibody, and the adsorbed plasma was still reactive, but the strength of the reaction was reduced by gel. A diagnosis of both cold and warm autoantibodies was confirmed, while all major clinically significant alloantibodies were excluded.

Based on these findings, prednisone was discontinued, and the patient was treated with rituximab 375 mg/m^2^ for a total of four doses. The prednisone course was limited to four days due to the discovery of cold antibodies as well as ongoing significant anemia. The patient began demonstrating signs of response after 2 doses of rituximab evidenced by decreasing transfusion requirements, improved hemoglobin stability, and improvement in hemolytic indices. Follow-up at 5 months after rituximab completion demonstrated ongoing hemoglobin stability with evidence of compensated hemolysis (Table 1).

## 4. Discussion

Mixed warm/cold autoimmune hemolytic anemia is a relatively rare entity and current evidence regarding optimal management of these cases is limited. Furthermore, there is considerable variability between sources in diagnostic criteria which further complicates the diagnosis and management of this pathology [1].

Previous studies have found that mixed warm/cold AIHA account for 6.5–8.3% of AIHA cases and approximately 50% of which are idiopathic without an identifiable secondary etiology [2, 3]. Interestingly, an association between mixed AIHA and SLE has been described; however, this was not observed in our case [4, 5]. Mayer et al. investigated 2194 patients with detected warm autoantibodies and found only 2 patients, less than 0.1%, had features consistent with mixed warm/cold AIHA which makes the true prevalence of this pathology difficult to ascertain [1].

The use of steroids in the management of this condition has been previously described with a mixed response, and in several cases, patients required splenectomy due to ongoing hemolysis (Table 2) [1, 4–18]. We conducted a literature review of case reports published and available on PubMed since the year 2000 with confirmed mixed AIHA (confirmed warm antibodies and cold agglutinins based on DAT) and their clinical course (Table 2). Additional case reports were excluded for unavailable clinical details [19, 20]. Rituximab, a monoclonal CD20 antibody, is another treatment that has recently been employed as an efficacious therapeutic strategy amongst warm and cold AIHA [21–24]. Rituximab has been described for the management of mixed AIHA, and in a case report, a patient responded with resolution of hemolysis after two courses of rituximab following initial management with prednisone [8]. Our study similarly demonstrated an initial response following one course of rituximab with stabilization in hemoglobin, and will continue to be observed for long-term remission.

In summary, mixed warm/cold AIHA represents a complex diagnosis, and the optimal management of these cases has not been well elucidated. Case series report largely limited early response following steroid therapy, and recent case reports suggest a promising response to rituximab. However, given the limited literature surrounding this topic, additional investigations would be required to further elucidate the optimal management of this pathology.

## Figures and Tables

**Table 1:** Laboratory values at presentation, pre- and post-prednisone, and pre- and post-rituximab.

Value	At presentation	Preprednisone^*∗*^	Postprednisone	Prerituximab^*∗∗*^	Postrituximab	5 months postrituximab	Reference range
HGB	59	44	74	69	95	99	4.0–11.0 × 109/L
Total bilirubin	37	47	41	46	57	21	3–17 *μ*mol/L
LDH	1347	1300	1467	1489	1584	692	84–246 U/L
Haptoglobin	<0.08	<0.08	Not tested	<0.10	<0.10	<0.10	0.30–2.00 g/L

^*∗*^Prednisone 1 mg/kg for four doses, ^*∗∗*^Rituximab 375 mg/m^2^ weekly for four doses.

**Table 2:** Case reports of mixed autoimmune hemolytic anemia and their clinical course.

Study	Year of publication [ref]	Number of patients	Associated condition	Treatment employed	Response
Mayer et al	2008 [1]	2	SLE (2)	(1) No treatment (2) Prednisone (30 mg/day) and azathioprine (100 mg/day)	(1) Did not require treatment (2) Response after unclear duration

Wondergem et al	2006 [4]	1	SLE	Prednisone (1 mg/kg/day unknown duration), IVIG	Persistent hemolysis despite prednisone, response to IVIG and subsequent tapering of steroids with resolution of hemolysis

Sudha Reddy et al	2011 [5]	1	Unknown	Prednisolone (2 mg/kg/day for 4 weeks), tapered to alternating day steroids	Response, tapered to corticosteroids on alternating days

Tanaka et al	2006 [6]	1	Following chicken pox infection	Methylprednisone (1000 mg/day unclear duration), prednisolone (60 mg/day for 4 weeks tapered to 10 mg/daily)	Initial response to methylprednisolone, worsening thrombocytopenia following taper treated with reinitiation of methylprednisone and IVIG (400 mg/day 3 days) with stabilization, recurrent hemolysis requiring reinitiation of prednisone

Win et al	2007 [7]	2	(1) Unknown (2) Splenic T-cell angioimmunoblastic non-Hodgkin's lymphoma	(1) Prednisone (40 mg/day unclear duration) and IVIG (0.4 mg/kg for 5 days) (2) prednisone (60 mg/kg, increased to 100 mg/kg unclear duration) and IVIG (2 mg/kg for 2 days); VCP (cyclophosphamide 750 mg/m^2^, vincristine 2 mg and prednisone 50 mg unclear number of cycles); rituximab (325 mg/m^2^ for 2 weeks); splenectomy	(1) Response with stabilization in hemoglobin (2) No response to steroids/IVIG, chemotherapy or rituximab; response to splenectomy with stabilization in hemoglobin

Morselli et al	2002 [8]	1	Unknown	Prednisone (1 mg/kg/day) with taper; over three weeks to 25 mg/daily, rituximab (325 mg/m2 for 2 weekly courses)	Response to initial steroid treatment with following taper, recurrence in hemolysis with response and stabilization in hemoglobin with 2 cycles of rituximab

Qiao et al	2016 [9]	1	Primary Sjorgen syndrome	Methylprednisone (40 mg/day unknown duration) transitioned to pulse methylprednisone (1000 mg/day for 3 days) followed by prednisone 50 mg/day (unknown duration), patient received G-CSF and cyclophosphamide 0.2 mg every other day to treat concomitant agranulocytosis	Complete response with resolution of hemolysis

Imataki et al	2020 [10]	1	Idiopathic, prior autologous stem cell transplant for DLBCL	Prednisolone (1 mg/kg for unknown duration)	Progressive hemolysis without response

Hirano et al	2016 [11]	1	SLE	Prednisolone (2 mg/kg for 2 months and subsequent taper), 2 courses of methylprednisone pulse (unknown dosage/duration) MMF (1000 mg/day tapered 500 mg/day for unknown duration)	Response with stabilization in hemoglobin

Scaramucci et al	2005 [12]	1	Idiopathic	Prednisone (1 mg/kg/day unknown duration with taper), rituximab (375 mg/m^2^ four weekly courses)	Response with prednisone with relapse following taper, complete response to rituximab

Elharake et al	2019 [13]	1	EBV PCR positive	Methylprednisone (unknown dosage and duration), plasma exchange	Response with hemoglobin stability

Haller et al	2009 [14]	1	Post liver transplant without evidence of alloantibodies, EBV and CMV positive	IV methylprednisone (1 mg/kg QID for 7 days) transitioned to prednisolone (1 mg/kg daily), rituximab (325 mg/m^2^ for 4 weekly courses), IVIG (0.5 g/kg, dose for 3 weekly doses), prednisolone tapered to 2.5 mg daily over 6 months and subsequently continued as GVHD prophylaxis	No response to initial methylprednisone, response to rituximab with stabilization in hemoglobin and resolution of transfusion requirements

Zhang et al	2012 [15]	1	Hodgkin lymphoma	IV methylprednisone (unclear dose and duration), 1 cycle of rituximab (unclear dose)	No response to initial methylprednisone, response to rituximab with hemoglobin stabilization

Rai et al	2017 [16]	2	Idiopathic	Both cases received corticosteroids (unknown medication, dose and duration)	Both had response to corticosteroid therapy with improvement in hemolysis however long term follow up unknown

Gupta et al	2011 [17]	1	Idiopathic	IV methylprednisone (unknown dose and duration), plasmapheresis for 7 daily doses, rituximab (375 mg/m^2^ for four weekly doses)	No response to initial corticosteroid or plasmapheresis, stabilization in hemoglobin following initiation of rituximab and transfusion independence

Webster et al	2004 [18]	1	Idiopathic	Cyclophosphamide 50 mg daily and prednisone 10 mg alternating days for 1 month; rituximab 700 mg IV for 4 weekly doses	Discontinued cyclophosphamide/prednisone due to side effects and progressive hemolysis, response to rituximab with resolution of transfusion requirements and hemoglobin stabilization

DLBCL: diffuse large b-cell lymphoma, MMF: mycophenolate mofetil VCP: vincristine, cyclophosphamide, and prednisone.

## Data Availability

The data used to support the findings of this study are available from the corresponding author upon reasonable request.
